# Building Linked Open Data towards integration of biomedical scientific literature with DBpedia

**DOI:** 10.1186/2041-1480-4-8

**Published:** 2013-03-13

**Authors:** Yasunori Yamamoto, Atsuko Yamaguchi, Akinori Yonezawa

**Affiliations:** 1 Database Center for Life Science, Faculty of Engineering Bldg. 12, The University of Tokyo, 2-11-16, Yayoi, Bunkyo-ku, Tokyo, Japan

## Abstract

**Background:**

There is a growing need for efficient and integrated access to databases provided by diverse institutions. Using a linked data design pattern allows the diverse data on the Internet to be linked effectively and accessed efficiently by computers. Previously, we developed the Allie database, which stores pairs of abbreviations and long forms (LFs, or expanded forms) used in the life sciences. LFs define the semantics of abbreviations, and Allie provides a Web-based search service for researchers to look up the LF of an unfamiliar abbreviation. This service encounters two problems. First, it does not display each LF’s definition, which could help the user to disambiguate and learn the abbreviations more easily. Furthermore, there are too many LFs for us to prepare a full dictionary from scratch. On the other hand, DBpedia has made the contents of Wikipedia available in the Resource Description Framework (RDF), which is expected to contain a significant number of entries corresponding to LFs. Therefore, linking the Allie LFs to DBpedia entries may present a solution to the Allie’s problems. This requires a method that is capable of matching large numbers of string pairs within a reasonable period of time because Allie and DBpedia are frequently updated.

**Results:**

We built a Linked Open Data set that links LFs to DBpedia titles by applying key collision methods (*i.e.*, fingerprint and n-gram fingerprint) to their literals, which are simple approximate string-matching methods. In addition, we used UMLS resources to normalise the life science terms. As a result, combining the key collision methods with the domain-specific resources performed best, and 44,027 LFs have links to DBpedia titles. We manually evaluated the accuracy of the string matching by randomly sampling 1200 LFs, and our approach achieved an F-measure of 0.98. In addition, our experiments revealed the following. (1) Performances were similar independently from the frequency of the LFs in MEDLINE. (2) There is a relationship (r^2^ = 0.96, P < 0.01) between the occurrence frequencies of LFs in MEDLINE and their presence probabilities in DBpedia titles.

**Conclusions:**

The obtained results help Allie users locate the correct LFs. Because the methods are computationally simple and yield a high performance and because the most frequently used LFs in MEDLINE appear more often in DBpedia titles, we can continually and reasonably update the linked dataset to reflect the latest publications and additions to DBpedia. Joining LFs between scientific literature and DBpedia enables cross-resource exploration for mutual benefits.

## Background

### Linked data in bioinformatics and systems biology

Because of the rapid developments in the life sciences and the large amounts of open data available on the Internet, a rising number of databases are being released. Currently, 1,380 databases are listed on the 2012 NAR Database Summary Paper Alphabetic List [1], which have been carefully selected by Nucleic Acids Research editors [2]. A single organisation or institution cannot build or maintain all of these databases, but none of the databases is all-encompassing; each researcher must identify multiple databases that are relevant to his/her research and learn how to use them to look up specific, designated entries. In such a situation, linking related entries would make the research process more efficient. For example, a systems biology researcher would need to access databases of chemical biology and drug data to discover a new drug. This type of interdisciplinary work has successfully addressed various complicated research challenges [3]. Linking related research outcomes beyond field-specific boundaries would further the progress of interdisciplinary studies.

### The scientific literature, the entities as abbreviations and long-forms

To link heterogeneous databases and provide users with access in an integrated manner, publishing datasets following the linked data design pattern [4] has increasing appeal to database developers and users. One reason for this is that it utilises well-known open standards, such as the Resource Description Framework (RDF) [5] or Hypertext Transfer Protocol (HTTP). Within this context, we decided to make our abbreviation database, Allie, downloadable in RDF format. The Allie database stores life science abbreviations and their long forms (LFs, or expanded forms) [6]. We have been providing a Web interface to lookup the candidate LFs of given abbreviations since 2008, and we began to provide a SPARQL endpoint in 2011. The dataset is updated monthly to keep up with the latest publications, and it is publicly available free of charge. Our motivation for developing this database is based on the following facts: Life science researchers often have difficulty in understanding papers that are outside their area of expertise partly because abbreviations are commonly used in those papers and polysemous ones appear frequently. We use the ALICE tool [7] to extract pairs of abbreviations and their corresponding LFs from the entire MEDLINE database. Allie stores each pair with its associated PubMed IDs such that users can easily find its provenance. Although Allie displays several contexts in which each pair is used (*e.g.*, bibliographic data, co-occurring abbreviations, and a main research area), Allie does not define the LF itself. We assumed that providing a description of each LF would be beneficial to the users; however, creating descriptions for every LF from scratch is impractical at our institution.

### Wikipedia as background information

Wikipedia [8] is an open, collaboratively developed encyclopedia project and the largest, most popular general reference work on the Internet [9]; it is expected to contain a considerable number of entries corresponding to LFs. Therefore, using this content as a reference for the descriptions of each LF may provide a solution. Furthermore, there are advantages to using Wikipedia. First, its content is licensed under Creative Commons Attribution Share-Alike 3.0, and we can freely obtain the entire dataset. Second, in the form of DBpedia [10], Wikipedia is also used as a hub for the Linked Open Data (LOD) cloud [11]; Allie users can access related information that would otherwise be difficult to find by following a series of links from an abbreviation-LF pair. The LOD cloud is an outcome of the Linking Open Data project [12], which is a grassroots community effort founded in 2007 to identify datasets available under open licenses, re-publish them in RDF on the Web, and interlink them with each other. The latest version contains 295 datasets that consist of 31 billion RDF triples, which are interlinked by approximately 504 million RDF links [13]. In the cloud, 42 datasets are categorised as life science datasets. Third, the titles of Wikipedia entries can be used as a gold standard set to normalise the lexical variants of LFs because they are the results of the collaborative knowledge building.

Following these discussions, we decided to link Allie LFs to DBpedia entries. Although this task appears rather straightforward, as is frequently the case with natural language processing, it is difficult to cope with the lexical variants. In addition, the numbers of LFs and Wikipedia entries are large (1,768,718 LFs and 8,826,375 entries, respectively), and new abbreviations and Wikipedia entries are continually generated independently. Integration of literature and Wikipedia enables researchers at different levels of expertise to better exploit either resource by linking established knowledge with latest research results. Therefore, the linking process must be automated and completed within a reasonable time. Thus, our task consists of checking whether each LF matches any of the DBpedia titles, efficiently taking their lexical variants into consideration and making a link using the *owl: sameAs* property, which is a built-in Web Ontology Language (OWL) property.

To make links effectively and efficiently between Allie and DBpedia, we took a strategy consisting of two approaches, that is, approximate string matching and dictionary-based term normalisation to take domain knowledge into consideration. We used Google Refine [14] for the former, which is a tool used to clean data that can also be used for data reconciliation. Google Refine has several functions, including data distribution analysis and values editing. In addition, it provides clustering functionality within the values of a column to help users identify lexical variants or typographical errors, and the Java source code is available for free. We can choose several well-studied clustering algorithms and approximate string-matching methods for use in this clustering [15]. For the latter, we used the Unified Medical Language System (UMLS) dictionaries and a tool (UMLS resources) [16] to map an inflectional form to its corresponding base form (*i.e.*, normalisation).

Our contributions to the biomedical semantics community include publishing a Linked Open Data set for the life sciences, demonstrating a method of efficiently and effectively constructing links between large and rapidly changing datasets, and enriching the Allie service. In addition, we confirmed that there is a statistically supported relationship between abbreviation-LF usage in the life science literature and the probability of its appearance in DBpedia titles.

## Results

To see a relationship between the occurrence frequencies of LFs in MEDLINE and the probabilities of their appearance in DBpedia titles, we split the LFs used in the evaluation into 12 bins according to the numbers of their occurrences in MEDLINE. Then, we sampled 100 LFs randomly from each bin; therefore, we manually evaluated 1200 randomly sampled LFs.

**Figure 1:**
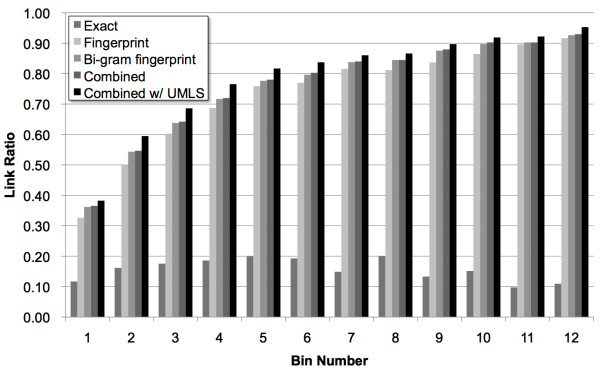
**Link results. **Link results for five methods/conditions: exact match, the key collision methods of fingerprint and bi-gram fingerprint, and the combined method with and without using the UMLS resources. The link ratio indicates the number of LFs with a link to their corresponding DBpedia titles divided by the total number of LFs at each bin.

Our experiments resulted in two sets of data: a set of link (match) ratios between the LFs and DBpedia titles for each bin and a set of match performance results. A link ratio means here that the number of the generated links divided by the total number of LFs in a bin. The former set demonstrates the extent to which our methods can produce links between LFs and DBpedia titles (these may include false links), and the latter set describes their accuracy; Figure 1 and Table 1 indicate these values, respectively.

**Table 1:** F-measures for each bin and method

**Bin**	**Exact match**	**Fingerprint**	**Bi-gram fingerprint**	**Combined**
1	0.80	0.98	0.99	0.99
2	0.83	0.92	0.94	0.96
3	0.86	0.97	0.98	0.99
4	0.80	0.96	0.95	0.99
5	0.85	0.96	0.98	0.98
6	0.88	0.94	0.97	0.97
7	0.88	0.97	0.99	0.99
8	0.92	0.96	0.97	0.97
9	0.86	0.97	0.97	0.98
10	0.93	0.98	0.98	1.00
11	0.90	0.97	0.98	0.99
12	0.91	0.98	0.98	0.99

Each F-measure expresses the accuracy of the string match. Note that all the data have been obtained using the UMLS-based term normalisation pre-processes, and these do not indicate how many LFs have links to their corresponding DBpedia titles.

Figure 1 illustrates the link ratios for each bin and each method. For the results of the exact match, fingerprint, and bi-gram fingerprint methods, the UMLS resources were not used; for the combined method, the results with and without the UMLS resources are shown. The results indicate that as the number of occurrences of an LF in MEDLINE increases, the link ratio also increases, with the exception of the exact match method. This outcome suggests that approximate string-matching methods (*i.e.*, the key collision methods of fingerprint and bi-gram fingerprint) significantly contribute to the increase in link ratios. In addition, combining the key collision methods also improves the ratios, and using the UMLS resources is effective for all the bins. Consequently, the best link ratios can be obtained with the combined method and using the UMLS resources. Note that we opted for bi-gram for the n-gram fingerprint method subsequent to our preliminary experiments. In addition, we also experimented the case of using the UMLS resources only (*i.e.*, the exact match method with the UMLS resources), but the result was worse than that of the combined method.

Table 1 presents the F-measures for each bin and method. This indicates that the match performance is high (from 0.92 to 1.00) for all of the bins and methods, with the exception of the exact match (from 0.80 to 0.93). Table 2 indicates the numbers of false negatives, false positives, and true positives. Here, we identify a false negative if an LF does not have a link to its corresponding DBpedia title (where there should be a link) and a false positive if an LF is erroneously linked to DBpedia titles.

**Table 2:** The numbers of false negatives (NPs), false positives (FPs), and true positives (TPs) for each method

	**FN**	**FP**	**TP**
Exact match	221	2	773
Fingerprint	59	9	928
Bi-gram fingerprint	40	9	945
Combined	28	4	967

Note that all of the data have been obtained using the UMLS-based term normalisation pre-processes.

These results indicate that the lower link ratios of the less frequently appearing LFs are not attributable to the drawbacks of the methods that were used; rather, the LFs that do not have a link to DBpedia do not have corresponding DBpedia titles.

We investigated the relationship between the occurrence frequencies of LFs in MEDLINE and their probabilities of presence in DBpedia titles. As a result, there is a linear relationship (r^2^ = 0.96, P < 0.01) between the occurrence frequencies of LFs and the negative logarithm of their probabilities of absence from DBpedia titles. More precisely, the relationship obtained based on simple linear regression analysis is

$$-\text{log}\left(1-y\right)=0.2x+0.92,$$

where x is the frequency-based bin number of an LF and y is the probability of its presence in DBpedia titles. In other words, the equation is

$$1-y=\text{exp}-0.2\left(x+4.6\right).$$

The detailed data and calculations used to obtain this result are available in the Additional file 1. Note that because the frequency ranges of bins 1 and 2 are different from those of bins 3 to 11, we replaced them with a single bin whose link ratio is the average of those of the two bins. The range of bin 12 is also different, but the numbers of the LFs become marginal if we split the bin, and we believe that the effect of using that bin is insignificant toward obtaining the relationship. We then reassigned the bin numbers from 0 to 10 (*i.e.*, the former bins 1 and 2 become bin 0, bin 3 becomes bin 1, and so on).

## Discussion

Using the two simplest approximate string-matching methods and the domain-specific resources, we determined that our hypothesis was supported by positive results. The key collision methods that we used are simpler and faster than other approximate string-matching methods, such as the Dice coefficient [17], the Jaro-Winkler distance [18], or the Levenshtein distance [19]. These methods are more flexible and are capable of finding less similar but possibly related strings; however, these methods also raise the probability of false positives and are more computationally intensive. Therefore, the methods can be used most effectively if there is a dramatic increase in the matching performance. We used the Levenshtein distance, but we terminated the process before completion after it had continued for more than three days. In contrast, the key collision methods took less than ten minutes under the same circumstances and machine environment.

Although the matching performance was high, our future research will benefit from a few alterations. There are some typical causes of false negatives and false positives. Three issues commonly result in false negatives (each example denotes a pair of an LF in Allie and its corresponding DBpedia title):

a. Presence/absence of an additive term

Examples include *c-fosproteinand C-Fos*, *bronchiolitis obliteranssyndrome* and *Bronchiolitis obliterans*, or *natural killer T* and *Natural Killer Tcell*.

b. Abbreviation/LF

Examples include *Programmed death-1* and *PD-1*, *reverse transcriptionPCR* and *Reverse transcriptionpolymerase chain reaction*, or *endothelial NOsynthase* and *Endothelial NOS*.

c. Variant/synonym

     Examples include *cytochrome P4501A1* and        *Cytochrome P450,family 1, member A1*;         *epithelial*  *tomesenchymal transition* and         *Epithelial mesenchymal transition*; or         *transoesophagealechocardiography* and         *Transesophagealechocardiogram*.

To resolve the first issue, we require a simple dictionary of these domain-specific additive terms (for proteins, syndromes, or cells). This dictionary can be used to match the last word of a compound; if there is a match for a term (compound), the matched word can be ignored. The second issue is caused by the difference between an abbreviation and its LF and can be resolved using Allie’s dictionary. For example, there is a pair of *PD-1* and *programmed death-1* in Allie; therefore, the both can be linked. However, we must ensure that the *PD-1* used as a DBpedia title definitely describes *programmed death-1* because this may be a polysemous word. The third issue is caused by synonymy, and the UMLS resources can partially help if they can successfully enumerate the relevant synonyms.

In addition, DBpedia data (other than titles) can be used to address these issues. For example, the predicates http://dbpedia.org/ontology/wikiPageDisambiguates and http://dbpedia.org/ontology/wikiPageRedirects denote that their subjects and objects have synonymous relationships one another. Therefore, if there is a triple stating that *PD-1* is a synonym for *programmed death-1*, the problem described above can be solved.

Although there are far fewer false positives than false negatives, the following examples are relevant to this domain. The first example is *mitogen-activated protein kinase kinase* and *Mitogen-activated protein kinases*. In general, it is considered erroneous if a term appears consecutively multiple times, and the key collision methods do not distinguish the former from the latter. The second example is *RNA polymerase II* and *Rna polymerase iii*. In this case, the methods do not distinguish between the two words *II* and *iii*, in which the difference is the number of appearances of a repeated letter. These cases require unique fixes.

As for our choice of DBpedia, some of the 42 life science datasets in the LOD cloud might be more relevant to the Allie database. However, Allie covers all of the life science research areas as MEDLINE does and needs the description of each LF; there is not such one in the 42 datasets. Furthermore, as DBpedia is the hub, once successfully linking Allie to it, we can access to the relevant datasets via DBpedia.

Regarding constructing linked data, we can use an integrated development environment called Silk [20] to build linked data between any two sets of RDF data for which SPARQL endpoints have been provided. Although Silk provides several types of string-matching methods, such as the Jaccard similarity coefficient, the Jaro distance, or the Levenshtein distance, key collision methods are not included. In addition, the current version (Version 2.5.3) of Silk does not provide a way to use external resources to match a pair of strings, such as the UMLS resources.

First, we assumed that the more frequently the LFs appear in MEDLINE, the more likely they are to appear in DBpedia as titles. Once this assumption has been supported, we can expect that if an LF will be used more frequently in literature whose bibliographic data are in MEDLINE, the LF will also be used as a title in DBpedia. We believe that this feature is reasonable for use as an enhancement for Allie because the LFs that more researchers want to look up are more likely to have their corresponding titles in DBpedia.

Next, we confirmed that our assumption was supported based on the relationship between the occurrence frequencies of LFs in MEDLINE and their presence probabilities from DBpedia titles. This result can be understood to indicate that the likelihood of an LF appearing in the DBpedia titles is proportional to the absence probabilities of the LFs in DBpedia titles whose occurrence frequencies in MEDLINE are the same as that of the LF. Therefore, if an LF is continually used in the life science literature, it is likely to appear in the DBpedia titles.

We set the threshold cut-off to 10 for the occurrence frequencies of LFs. This might be too high, and we need to investigate cases below it. Our preliminary survey indicated that more false negatives occurred owing to the case c (variant/synonym) mentioned above.

## Conclusions

First, we showed that the LFs that are most frequently used in MEDLINE titles or abstracts are more likely to appear as titles in DBpedia; LFs that do not currently have a link to DBpedia titles are expected to obtain a link if they are used more frequently in MEDLINE. This finding yields useful knowledge for Allie users.

Second, we proposed an effective linked data-building process that uses the two key collision methods (*i.e.*, fingerprint and bi-gram fingerprint) and the UMLS resources, based on our attempt to link LFs in the Allie database to DBpedia titles. We performed the experimental comparisons of exact match, fingerprint, bi-gram fingerprint, and the method combining the two key collision methods with the UMLS-based pre-processing. The results demonstrate that of the four methods, the combined method performed best, yielding a very high performance with an F-measure of 0.98. Because the key collision methods are known to be much faster than other approximate string-matching methods, using them is an effective way to build linked data, even for very large datasets.

Together with the obtained relationship, this outcome is promising as a means of managing the rapid growth of data in MEDLINE and DBpedia; it allows us to continually update the linked dataset.

We analysed examples of false negatives and found that they exhibit certain typical patterns, which will be explored in the future. From the perspective of Linked Open Data, we plan to place the newly acquired links on the results page of the Allie search service and add them to the Allie RDF datasets, which are publicly downloadable for free and accessible at the SPARQL endpoint [21].

## Methods

### Data

a. Allie

Currently, the Allie database has 1,768,718 LFs, which were automatically extracted from MEDLINE (titles and abstracts; more than 20 million entries) by ALICE. Although ALICE’s performance is fairly high (a recall of 95% and a precision of 97% on randomly selected MEDLINE data), erroneous expressions are included, especially when the number of their appearances in MEDLINE is low. Therefore, we decided to use expressions that appear more than 10 times in MEDLINE as a target dataset to be linked to DBpedia. As a result, the number of the extracted LFs is 91,573.

b. DBpedia

We used the English titles of DBpedia version 3.7 in N-Triples (labels_en.nt.bz2). There are 8,826,375 titles.

c. UMLS

   UMLS is a set of files and software that brings       many health and biomedical vocabularies and       standards together developed and is maintained       by the U.S. National Library of Medicine       (NLM). To absorb some of the lexical variants       in the LFs and DBpedia titles, we used the       UMLS SPECIALIST Lexicon and the lvg norm       tool. For the Lexicon, we used the ‘Agreement       and Inflection’ and the ‘Spelling Variants’ files       to map each term to its basic form. If a basic       form is found in which the inflectional form       exactly matches an LF or a DBpedia title, it is       replaced with the basic form. The lvg tool is       suitable for this purpose, but our survey       showed that it fails to normalise some terms       that can be handled with the Lexicon mapping.

### String matching

We used two key collision methods for approximate string matching: fingerprint and n-gram fingerprint [15]. In addition, as a baseline to compare the results, exact string matching was used. The key collision methods are explained well in [15]; a brief explanation is provided here. For each string, its alternative expression (key) is generated and matched against another string’s key to determine whether they are identical. The key collision methods are fast: their computational complexity is linear in the number of values processed. The difference between fingerprint and n-gram fingerprint is the way in which keys are generated. The former method consists of a series of string manipulation processes to generate a key for each string, such as splitting the string into whitespace-separated tokens, sorting the tokens and removing duplicates, and joining the tokens back together. This method absorbs word order variations and is the least likely to produce false positives. The latter method is similar to the fingerprint method, but instead of using whitespace-separated tokens, it uses n-grams in which the n (or the size in *chars* of the token) can be specified by the user. In our task, we use size two, based on a comparison of the results from the use of bi-gram and tri-gram.

### Experiments

To test our assumption (*i.e.*, the relationship between the numbers of appearance of LFs in MEDLINE and the probability of their appearance in DBpedia), we split the LF data into 12 bins according to their occurrence frequencies in MEDLINE (Table 3). For example, the LF *lentigo maligna melanoma* appears 63 times in MEDLINE and is therefore in bin 2 (whose range is 50 to 99). The number of unique LFs (non duplicate LFs) that reside in bin 2 is 10,293. For each bin, we then obtained the matching results between the LFs and the DBpedia entry titles in the four ways, that is, exact match, fingerprint, n-gram fingerprint, and combining these two key collision methods. In the fourth way, a link between an LF and a DBpedia entry title is accepted if the fingerprint or the n-gram fingerprint methods find a match; if the two methods find different matches between an LF and DBpedia titles, the shorter matched title is accepted. Furthermore, if both string lengths are the same, the one in which the ratio of the numbers of upper to lower case characters is closest to that of the LF is accepted. For each method, we also obtained results using the UMLS resources.

**Table 3:** Distribution of LF appearances in MEDLINE

**Bin number**	**Frequency range of appearances in MEDLINE**	**Number of unique LFs**
1	10 - 49	69 416
2	50 - 99	10 293
3	100 - 199	5 691
4	200 - 299	2 030
5	300 - 399	1 033
6	400 - 499	639
7	500 - 599	444
8	600 - 699	329
9	700 - 799	233
10	800 - 899	185
11	900 - 999	154
12	>= 1 000	1 126

The frequency range indicates that the number of appearances of an LF in MEDLINE falls within that range.

To evaluate the results, we randomly sampled 100 LFs per bin and manually checked whether each link was appropriate. Furthermore, for each LF without any links, we determined whether there was a corresponding DBpedia title by searching for any corresponding strings in Wikipedia using Google.

## Abbreviations

LF: Long form; RDF: Resource Description Framework; HTTP: Hypertext Transfer Protocol; UMLS: Unified Medical Language System.

## Competing interests

The authors declare that they have no competing interests.

## Authors’ contributions

YY designed this work, and ATY discussed the experiments and outcomes. AKY oversaw this work. All authors read and approved the final manuscript.

## Supplementary Material

Additional file 1 Supplementary Data. Click here for file
